# NRG1/PDGFC loop between fibroblasts and cancer cells drives paclitaxel resistance via ferroptosis suppression in breast cancer

**DOI:** 10.1038/s41420-025-02785-2

**Published:** 2025-11-10

**Authors:** Wan-Li Duan, Xue-Jie Wang, Li-Hui Gu, Ai Guo, Yi-Yue Ding, Ping Lin, Bao-Gang Zhang

**Affiliations:** 1 Medical Research Center, Shaoxing People’s Hospital, Shaoxing, Zhejiang Province China; 2 Department of Diagnostic Pathology, School of Basic Medical Sciences, Shandong Second Medical University, Weifang, China; 3 School of Medicine, Shaoxing University, Shaoxing, Zhejiang P.R. China; 4 Department of Pathology, Shaoxing People’s Hospital, Shaoxing, Zhejiang Province China

**Keywords:** Chemotherapy, Cell signalling

## Abstract

Breast cancer (BC) is one of the leading diseases that severely threaten women’s lives and health worldwide, with chemoresistance remaining a major challenge in its treatment. The tumor microenvironment, particularly cancer-associated fibroblasts (CAFs), plays a critical role in the chemoresistance of tumor cells, but the underlying mechanisms involved still require further exploration. This study aims to investigate the role and potential mechanisms of the positive feedback loop formed by CAF-derived NRG1 and BC cell-derived PDGFC in paclitaxel resistance. To this end, we isolated primary CAFs from BC patients and established co-culture systems with BC cell lines to observe the impact of CAFs on paclitaxel resistance in BC cells. Exogenous NRG1 and the knockdown of NRG1 in CAFs were used to reveal the regulatory role of CAF-derived NRG1 in paclitaxel resistance in BC cells. CCK-8 assay, transmission electron microscopy, MDA and GSH/GSSG content measurements, as well as JC-1 assay, were used to assess ferroptosis levels in BC cells. Additionally, exogenous PDGFC and co-culture systems were used to investigate the effects of tumor cell-derived PDGFC on fibroblasts. Using a BC ectopic xenograft mouse model, we investigated the regulatory role of NRG1 and PDGFC in paclitaxel resistance in vivo. Our results showed that CAF-derived NRG1 significantly promoted paclitaxel resistance and ferroptosis escape in BC cells, while the AKT inhibitor effectively suppressed this effect. Moreover, BC cell-derived PDGFC activated fibroblasts and induced their high expression of NRG1. These findings suggest that CAF-derived NRG1 enhances ferroptosis escape and paclitaxel resistance in BC cells through the AKT/mTOR pathway, while also inducing cancer cells to express high levels of PDGFC. In turn, cancer cell-derived PDGFC promotes fibroblast activation and high NRG1 expression, forming a positive feedback loop between NRG1 and PDGFC. This feedback loop ultimately results in a malignant cycle of paclitaxel resistance in BC.

## Introduction

The mechanisms underlying the development and progression of breast cancer (BC) are highly complex and diverse [1]. Despite significant advances in BC treatment in recent years, the chemoresistance of cancer cells remains a major obstacle in clinical management [1]. The emergence of chemoresistance not only significantly reduces patient survival rates but also poses substantial challenges to treatment. Chemoresistance is mediated by multiple mechanisms, including the genetic heterogeneity of tumor cells, altered intracellular drug metabolism, activation of drug efflux pumps, and inhibition of cell death signaling pathways [2]. Among these mechanisms, the tumor microenvironment (TME), particularly cancer-associated fibroblasts (CAFs), plays a pivotal role in promoting chemoresistance in tumor cells [3]. Extensive evidence indicates that CAFs interact with tumor cells through the secretion of various cytokines, chemokines, growth factors, and exosomes, which not only regulate tumor cell proliferation, migration, and invasion but also significantly enhance their resistance to radiotherapy and chemotherapy [4–6]. In addition to regulating the biological behavior of tumor cells, tumor cells can also “reprogram” fibroblasts in the microenvironment into CAFs that favor tumor progression. This process involves direct cell-cell interactions, secreted cytokines, and alterations in the extracellular matrix components [7]. The information exchange between tumor cells and fibroblasts may form a mutually reinforcing positive feedback loop, which plays a critical role in cell signaling by amplifying systemic responses through enhanced activation of specific signaling pathways [8, 9]. Further elucidating the molecular interactions between CAFs and tumor cells, and understanding how these interactions promote chemoresistance in BC, could provide valuable insights for targeting intercellular signaling pathways to improve the prognosis and therapeutic outcomes of BC patients.

Paclitaxel (PTX) is one of the most important chemotherapeutic drugs for BC and a key component of standard chemotherapy regimens [10]. It plays a crucial role in neoadjuvant chemotherapy, adjuvant chemotherapy, and the treatment of advanced BC [10]. In preoperative chemotherapy, PTX effectively reduces tumor size, thereby increasing the pathological complete response (pCR) rate [11]. In postoperative therapy, PTX significantly decreases the risk of recurrence [12]. Moreover, in the treatment of metastatic or recurrent BC, PTX markedly prolongs both progression-free survival (PFS) and overall survival (OS) [13]. However, despite its remarkable efficacy in BC treatment, the emergence of cancer cell resistance to paclitaxel severely limits its long-term therapeutic effectiveness.

Neuregulin 1 (NRG1), a multifunctional growth factor in the epidermal growth factor (EGF) family, binds to ErbB family receptors and activates downstream signaling pathways, thereby regulating various biological processes [14]. Studies have shown that paracrine NRG1 secreted by CAFs can bind to human epidermal growth factor receptors 2/3 (HER2/3) on tumor cells, enhancing anti-androgen resistance in prostate cancer [15]. In esophageal squamous cell carcinoma, NRG1 has been demonstrated to promote cancer cell proliferation, migration, and afatinib resistance [16]. Furthermore, CAFs-derived NRG1 has been shown to mediate trastuzumab resistance in BC cells via the HER3/AKT signaling pathway [17]. However, the role of CAFs-derived NRG1 in chemoresistance of BC and its underlying mechanisms remain unclear. Elucidating the regulatory effects of CAFs-derived NRG1 on paclitaxel (PTX) resistance in BC will provide a theoretical foundation for improving therapeutic strategies for BC.

Programmed cell death (PCD) is one of the primary mechanisms by which cells are eliminated during chemotherapy, chemotherapeutic agents exert their therapeutic effects by inducing programmed cell death in tumor cells [18, 19]. However, the development of chemoresistance is often associated with the evasion or inhibition of PCD by tumor cells [19]. Ferroptosis, a novel form of PCD, has become a hot topic in cancer research, and is characterized by iron-dependent lipid peroxidation leading to cell death [20]. Studies have shown that targeting ferroptosis-related pathways can restore the sensitivity of tumor cells to chemotherapeutic agents, thereby overcoming resistance.

Platelet-Derived Growth Factor C (PDGFC) is a secretory protein belonging to the platelet-derived growth factor (PDGF) family. It plays a critical role in physiological processes such as tissue development, wound healing, and angiogenesis, and is closely associated with various diseases, including cancer and fibrosis [21–23]. In the TME, elevated PDGFC expression promotes the transition of normal fibroblasts (NFs) into CAFs, thereby significantly influencing tumor growth, migration, invasion, and the development of drug resistance [23, 24]. Evidence indicates that PDGFC secreted by tumor cells facilitates the lung colonization of BC cells and activates pulmonary fibroblasts, promoting distant tumor dissemination and progression [23].

In this study, we utilized co-culture models, exogenous recombinant proteins, gene knockdown techniques, and a mouse ectopic xenograft model to uncover the regulatory role of CAF-derived NRG1 in PTX resistance of BC cells. Further analysis revealed that PDGFC secreted by BC cells forms a positive feedback loop with CAF-derived NRG1, which significantly promotes PTX resistance in BC. This finding highlights the potential therapeutic opportunity of targeting the NRG1/PDGFC to mitigate chemoresistance in BC.

## Results

### CAFs promote PTX resistance in BC cells

**Fig. 1:**
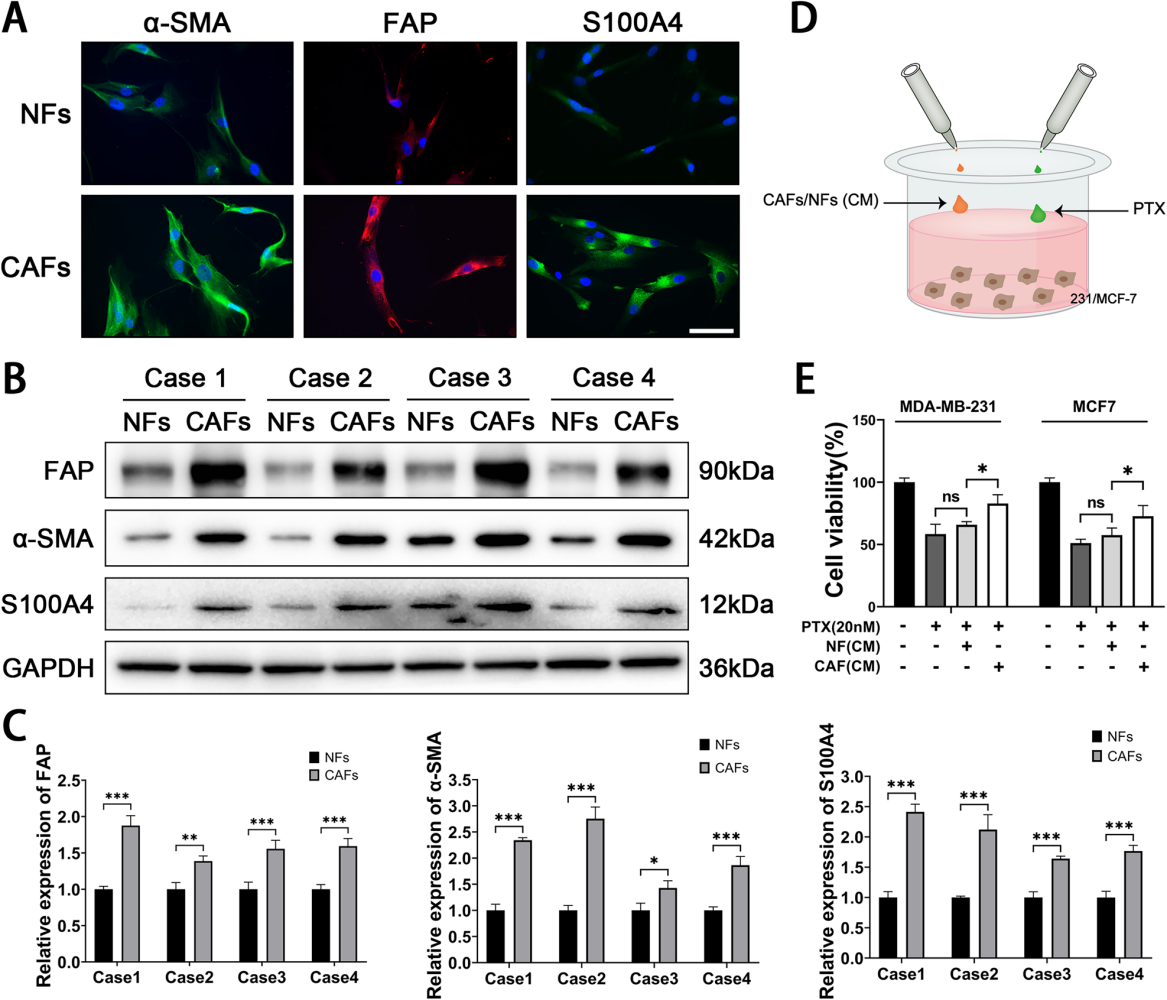
CAFs enhance PTX resistance in BC cells. **A** Immunofluorescence analysis of α-SMA, FAP, and S100A4 expression in fibroblasts. Scale bar = 50 μm. **B**, **C** Western blot analysis of FAP, α-SMA, and S100A4 expression in fibroblasts (*n* = 3). **D** Co-culture system established using CM from fibroblasts and BC cell lines. **E** CCK-8 assay to evaluate cell viability in BC cells (*n* = 3). Data are expressed as the mean ± S.D. for all panels: ns = no significance, *P < 0.05, **P < 0.01, ***P < 0.001.

To investigate the impact of CAFs on PTX resistance in BC cells, we isolated CAFs from BC tissues and NFs from paired adjacent normal tissues using the tissue block method (Fig. S1A, B). Immunofluorescence and western blot were performed to evaluate the expression levels of α-SMA, FAP, and S100A4 in the isolated CAFs and NFs. The results demonstrated that CAFs exhibited significantly higher expression of α-SMA, FAP, and S100A4 compared to NFs (Fig. 1A–C). Subsequently, co-culture systems were established using conditioned media (CM) from CAFs or NFs and BC cell lines, followed by the addition of PTX to assess the effects of CAFs(CM) on PTX resistance in BC cells. The CCK-8 assay results indicated that CM from CAFs significantly enhanced PTX resistance in BC cells compared to that from NFs (Fig. 1D, E). These results suggest that CAFs may promote PTX resistance in BC cells by secreting certain factors.

### CAF-derived NRG1 promotes PTX resistance in BC cells

**Fig. 2:**
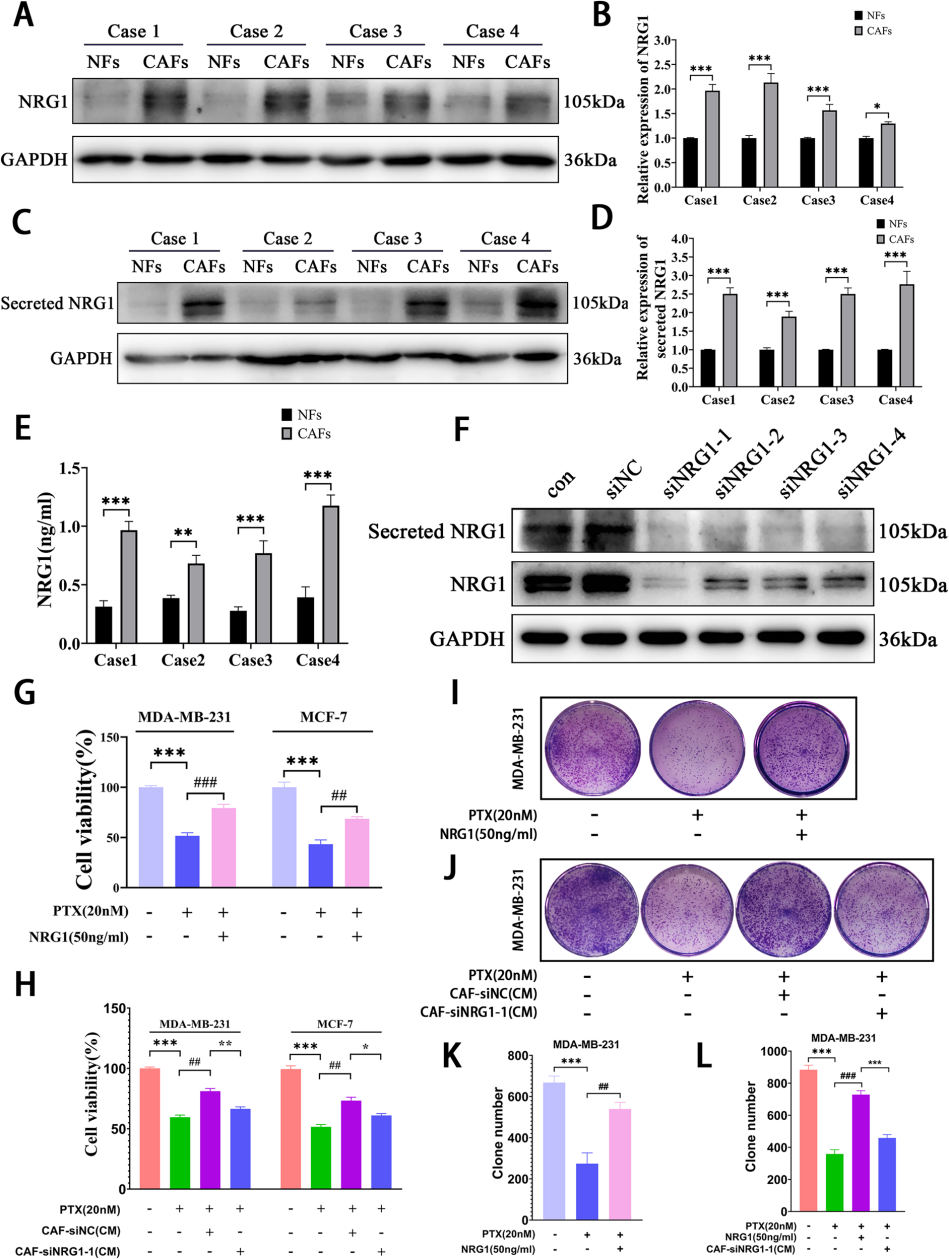
CAF-derived NRG1 promotes PTX resistance in BC cells. **A**, **B** Western blot analysis of NRG1 expression in CAFs and NFs (*n* = 3). **C**, **D** Western blot analysis of NRG1 content in the CM of CAFs and NFs, with NFs and CAFs from the original cell culture dishes used as internal controls (*n* = 3). **E** ELISA assay measuring NRG1 levels in the CM of CAFs and NFs (*n* = 3). **F** Western blot confirming the knockdown efficiency of NRG1 in CAFs by siRNA (*n* = 3). **G**, **H** CCK-8 assay evaluating the viability of BC cells (*n* = 3). **I**–**L** Colony-formation assay assessing the colony-forming ability of BC cells (*n* = 3). Data are expressed as the mean ± S.D. for all panels: *^, *^P < 0.05, **^, ##, **^P < 0.01, ***^,###,***^P < 0.001.

To further investigate the factors secreted by CAFs that enhance PTX resistance in BC cells, we reviewed the literature and found that CAFs promote anti-androgen resistance in prostate cancer and trastuzumab resistance in BC through the secretion of NRG1 [15, 17]. However, whether CAFs contribute to chemoresistance in BC by secreting NRG1 remains unexplored. First, we compared the expression levels of NRG1 in four pairs of CAFs and NFs, and the results showed that NRG1 expression was significantly higher in CAFs than in NFs (Fig. 2A, B). Similarly, the NRG1 content in secreted proteins from the CM of CAFs was higher than that of NFs (Fig. 2C, D). ELISA results further confirmed that NRG1 levels were elevated in CAFs(CM) compared to NFs(CM) (Fig. 2E). To verify whether exogenous NRG1 enhances PTX resistance in BC cells, we added recombinant NRG1 protein exogenously and found that it significantly enhanced PTX resistance in BC cells (Fig. 2G). However, when NRG1 was knocked down in CAFs, the ability of CAFs to promote PTX resistance in BC cells was attenuated (Figs. 2F, H, and S2A). Colony-formation assay showed that exogenous NRG1 increased the number of colonies after PTX treatment, whereas knocking down NRG1 in CAFs reduced the stimulatory effect of CAFs(CM) on colony formation (Figs. 2I–L and S2B–D). These findings suggest that CAF-derived NRG1 plays a critical role in promoting PTX resistance in BC cells.

### CAFs-derived NRG1 regulates ferroptosis sensitivity in BC cells

**Fig. 3:**
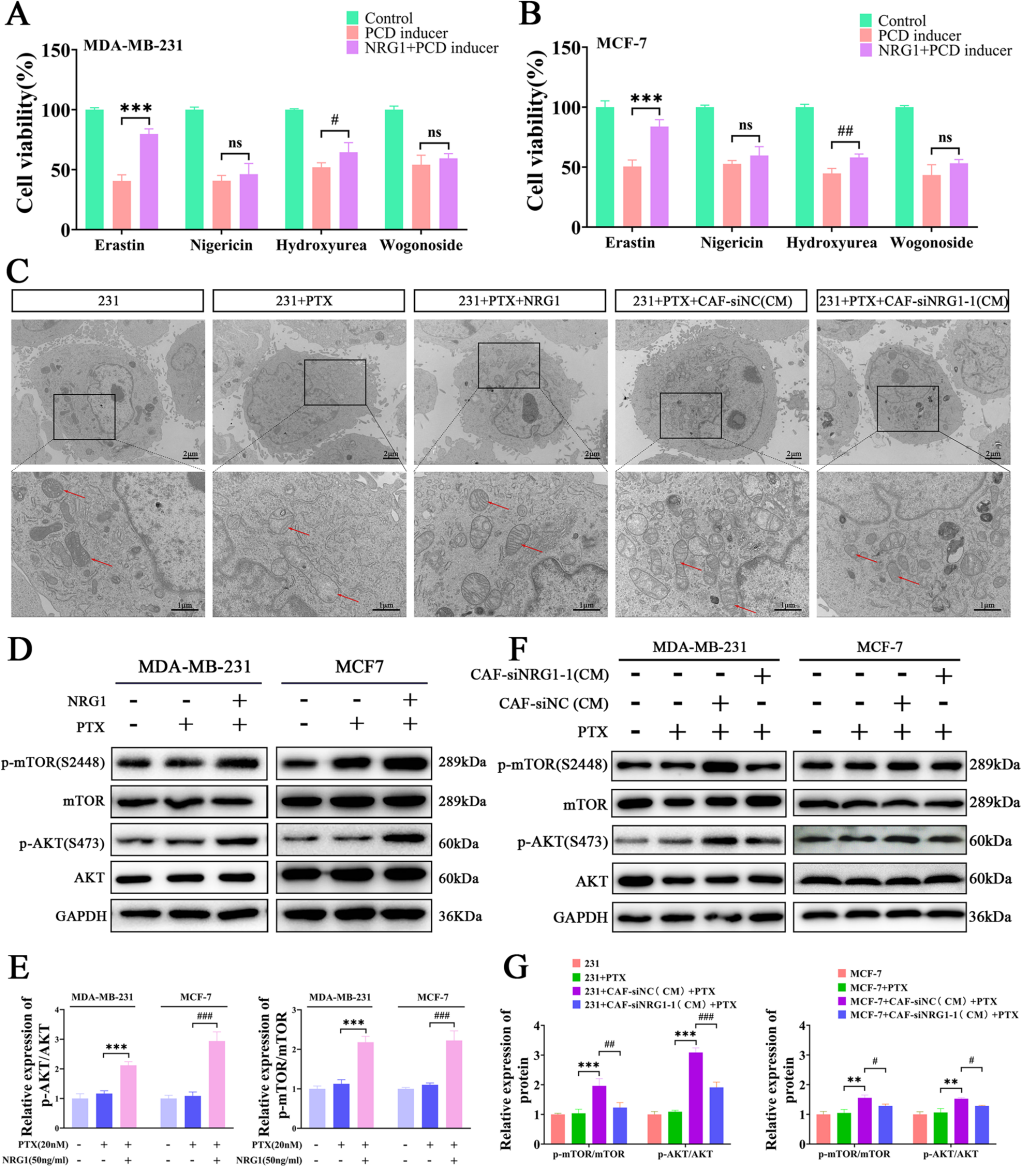
CAF-derived NRG1 promotes ferroptosis escape and AKT/mTOR pathway activation in BC cells. **A**, **B** CCK-8 assay to detect the inhibitory effect of NRG1 on cell death induced by Erastin, Nigericin, Hydroxyurea, and Wogonoside in BC cells (*n* = 3). **C** Transmission electron microscopy to observe mitochondrial damage in MDA-MB-231 cells. Scale bar = 2 μm (Zoom in = 1 μm). **D**, **E** Western blot to detect the effect of exogenous NRG1 on the AKT/mTOR pathway in BC cells (*n* = 3). **F**, **G** Western blot to observe the effect of CAF-derived NRG1 on the AKT/mTOR pathway in BC cells (*n* = 3). Data are expressed as the mean ± S.D. for all panels: ns = no significance, ^**#**^*P* < 0.05, **^, **##**^*P* < 0.01,^***,###^*P* < 0.001.

Induction of programmed cell death (PCD) is a key mechanism by which chemotherapy drugs kill cancer cells. We hypothesize that NRG1 may promote the PTX resistance of BC cells by inhibiting their PCD [18, 19]. To test this hypothesis, we used the ferroptosis inducer Erastin, necroptosis inducer Nigericin, apoptosis inducer Hydroxyurea, and autophagy inducer Wogonoside to induce cell death in BC cells, followed by the addition of recombinant NRG1 protein to observe its effect on cell viability. Our results showed that exogenous NRG1 significantly rescued cell death induced by ferroptosis and apoptosis inducers (Fig. 3A, B). Typical mitochondrial morphological changes in ferroptotic cells include membrane swelling, disappearance of inner membrane folding, and reduction of the matrix [25]. Transmission electron microscopy observations revealed that exogenous NRG1 significantly alleviated mitochondrial damage in MDA-MB-231 cells treated with PTX, whereas knockdown of NRG1 in CAFs significantly weakened the protective effect of CAFs(CM) on the mitochondria of BC cells (Fig. 3C). It is known that NRG1 activates the AKT/mTOR signaling pathway upon binding to ErbB receptors [26]. Therefore, we further investigated the effect of NRG1 on the AKT/mTOR signaling pathway in BC cells. Western blot results showed that exogenous NRG1 significantly activated the AKT/mTOR signaling in BC cells (Fig. 3D, E). Furthermore, CAFs(CM) also activated the AKT/mTOR signaling in BC cells, but this effect was significantly reduced upon knockdown of NRG1 in CAFs (Fig. 3F, G). These results suggest that CAF-derived NRG1 significantly decreases the sensitivity of BC cells to ferroptosis and activates the AKT/mTOR signaling pathway in cancer cells. However, whether NRG1 regulates ferroptosis sensitivity through the AKT/mTOR pathway in BC cells remains to be further explored.

### NRG1 promotes ferroptosis escape and PTX resistance through AKT/mTOR pathway in BC cells

**Fig. 4:**
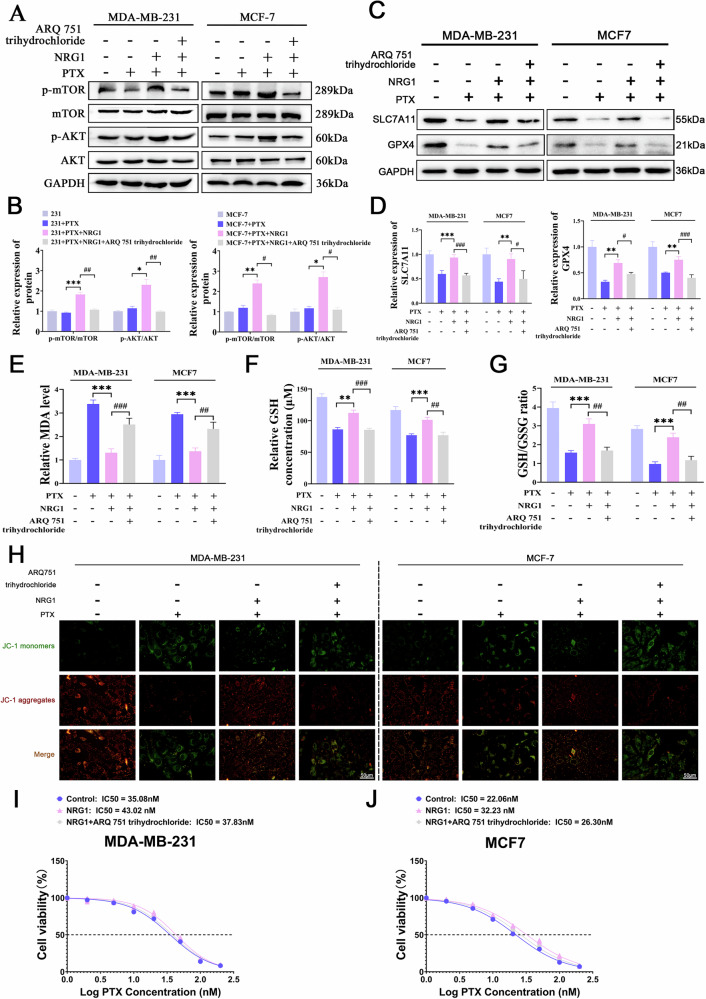
NRG1 promotes ferroptosis escape and PTX resistance through AKT/mTOR pathway in BC cells. (**A**, **B**) Western blot confirming the inhibitory effect of the AKT inhibitor ARQ751 trihydrochloride on the AKT/mTOR pathway in BC cells (*n* = 3). (**C**, **D**) Western blot detection of changes in SLC7A11 and GPX4 expression in BC cells (*n* = 3). **E** Measurement of MDA levels to assess lipid peroxidation in BC cells (*n* = 3). **F**, **G** Analysis of GSH content and the GSH/GSSG ratio in BC cells (*n* = 3). **H** JC-1 assay to evaluate changes in mitochondrial membrane potential in BC cells (*n* = 3). Scale bar = 50 μm. **I**, **J** CCK-8 assay used to determine the IC50 of PTX in BC cells (*n* = 3). Data are expressed as the mean ± S.D. for all panels: *^, **#**^*P* < 0.05, **^, **##**^*P* < 0.01^, ***,###^*P* < 0.001.

To further explore whether the effects of NRG1 on BC cells are mediated by the AKT/mTOR pathway, we utilized the AKT inhibitor ARQ751 trihydrochloride to suppress AKT/mTOR signaling in cancer cells. Western blot revealed that the AKT inhibitor significantly reversed NRG1-induced activation of AKT and mTOR phosphorylation (Fig. 4A, B). Moreover, NRG1 notably increased Bcl-2 levels while decreasing BAX and Cleaved-caspase3 levels, effects that were reversed by the AKT inhibitor (Fig. S3A–D). These findings align with our earlier observation that NRG1 provides resistance to apoptosis induced by apoptotic inducers. SLC7A11/GPX4 constitutes a critical pathway for ferroptosis resistance in cells and is regulated by AKT signaling [27, 28]. We hypothesized that NRG1 modulates the AKT/mTOR pathway to influence SLC7A11/GPX4 expression, thereby inhibiting ferroptosis in BC cells. Western blot demonstrated that NRG1 upregulated SLC7A11 and GPX4 expression, while the AKT inhibitor significantly reversed these effects (Fig. 4C, D). Malondialdehyde (MDA), an end product of lipid peroxidation, is a reliable marker of ferroptosis. Our findings showed that NRG1 significantly reduced MDA levels in PTX-treated cells, whereas the AKT inhibitor increased MDA levels (Fig. 4E). Additionally, glutathione (GSH) serves as a crucial antioxidant molecule, with its oxidized form being glutathione disulfide (GSSG). Measurement of GSH and GSH/GSSG ratios revealed that NRG1 significantly increased intracellular GSH levels and the GSH/GSSG ratio, effects reversed by the AKT inhibitor (Fig. 4F, G). Ferroptosis is often accompanied by mitochondrial damage, and JC-1 assays were conducted to assess mitochondrial membrane potential changes. The results indicated that NRG1 significantly enhanced mitochondrial membrane potential in PTX-treated cells, whereas the AKT inhibitor markedly reduced it (Fig. 4H). Furthermore, IC50 demonstrated that the AKT inhibitor significantly attenuated NRG1-mediated enhancement of PTX resistance in BC cells (Fig. 4I, J). These findings suggest that NRG1 mediates ferroptosis resistance and PTX resistance in BC cells through the regulation of the AKT/mTOR signaling pathway.

### PDGFC from BC cells forms a positive feedback loop with CAFs-derived NRG1

**Fig. 5:**
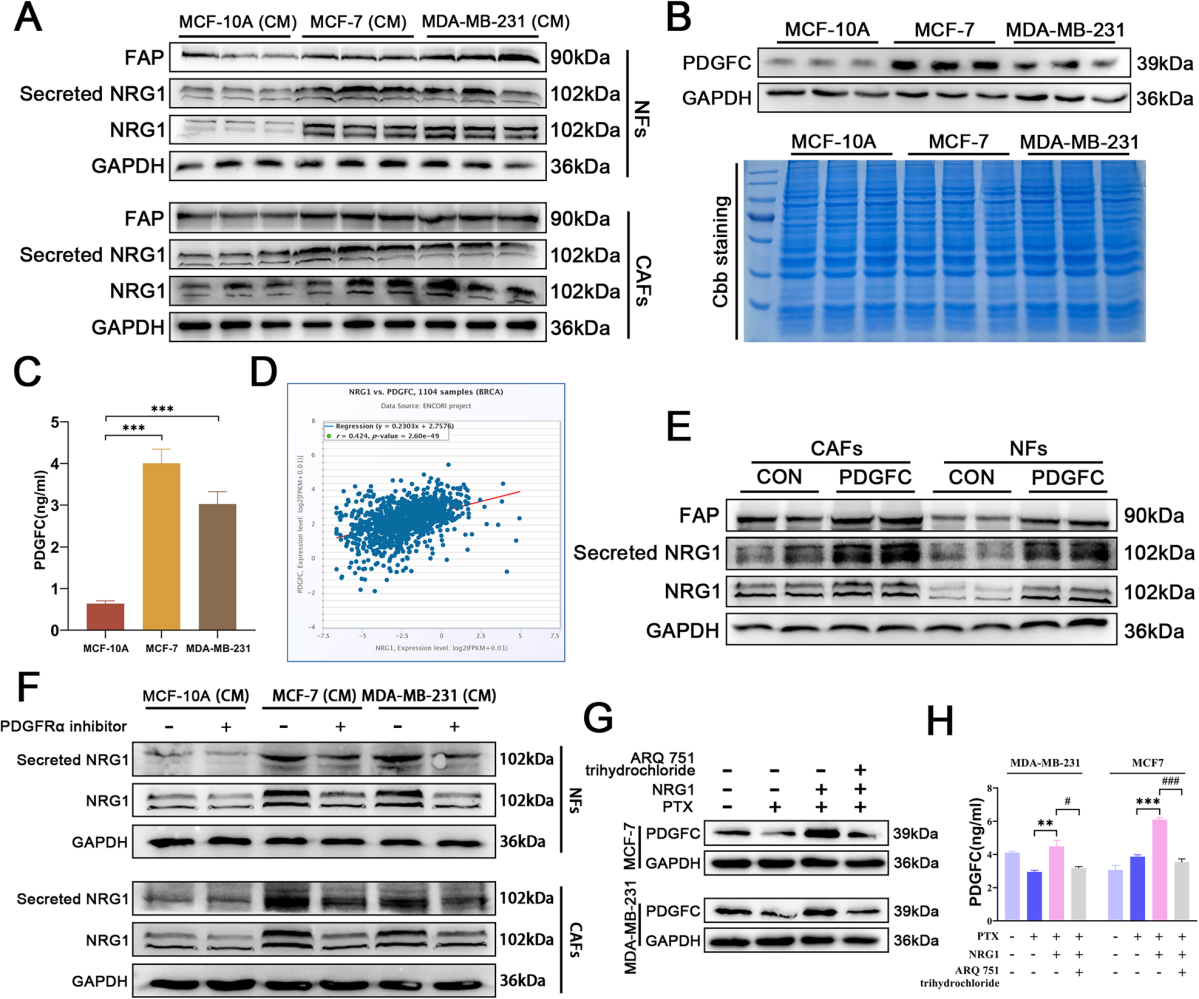
PDGFC from BC cells forms a positive feedback loop with CAFs-derived NRG1. **A** Western blot analysis of the effects of CM from normal breast epithelial cells and BC cells on FAP expression, as well as NRG1 secretion and expression in fibroblasts (*n* = 3). **B** Comparison of PDGFC expression levels in BC cells and normal breast epithelial cells using Western blot and Coomassie Brilliant Blue (Cbb) staining (*n* = 3). **C** ELISA analysis of PDGFC content in the CM of BC cells versus normal breast epithelial cells (*n* = 3). **D** ENCORI database analysis of the correlation between NRG1 and PDGFC in BC tissues. **E** Western blot analysis of FAP expression, and NRG1 expression and secretion in fibroblasts after the addition of exogenous PDGFC (*n* = 3). **F** Western blot evaluation of the inhibitory effect of PDGFRα inhibitors on NRG1 expression and secretion in fibroblasts (*n* = 3). **G** Western blot analysis of the effects of exogenous NRG1 and AKT inhibitors on PDGFC expression in BC cells (*n* = 3). **H** ELISA analysis of changes in PDGFC content in the CM of BC cells (*n* = 3). Data are expressed as the mean ± S.D. for all panels: ^**#**^*P* < 0.05, **P < 0.01, ^***, **###**^*P* < 0.001.

Positive feedback loops between tumor cells and CAFs play a crucial role in tumor progression. We aimed to investigate whether BC cells secrete specific factors that form a feedback loop with NRG1 derived from CAFs, thereby contributing to the vicious cycle of chemoresistance. First, we observed that the CM from BC cells significantly increased the expression and secretion of NRG1 in fibroblasts and enhanced the expression of FAP (Figs. 5A and S4A). Literature evidence suggests that activation of AKT signaling in cancer cells can upregulate PDGFC expression, and PDGFC secreted by tumor cells can activate fibroblasts [23, 29]. Thus, we hypothesized that PDGFC secreted by BC cells may form a positive feedback loop with NRG1 released by CAFs. To validate this hypothesis, we compared PDGFC levels in BC cells and normal breast epithelial cells via Western blot. The results showed significantly higher PDGFC expression in BC cells (Figs. 5B and S4B). ELISA further confirmed that the CM from cancer cells contained higher levels of PDGFC compared to that from normal epithelial cells (Fig. 5C). ENCORI database analysis revealed a positive correlation between NRG1 and PDGFC in BC tissues (Fig. 5D). When recombinant PDGFC protein was added exogenously, it significantly increased the expression and secretion of NRG1 in fibroblasts, as well as the expression of FAP (Figs. 5E and S4C). PDGFRα, the primary receptor for PDGFC [30], was inhibited using a PDGFRα inhibitor, which effectively suppressed the ability of cancer cell-CM to promote NRG1 expression in fibroblasts (Figs. 5F and S4D). Similarly, exogenous NRG1 significantly upregulated the expression and secretion of PDGFC in BC cells, and this effect was reversed by an AKT inhibitor (Figs. 5G, H and S4E). Collectively, these findings suggest that BC cells may establish a positive feedback loop by secreting PDGFC and stimulating CAF-derived NRG1, thereby promoting BC progression and chemoresistance.

### NRG1 and PDGFC promote PTX resistance in BC in vivo

**Fig. 6:**
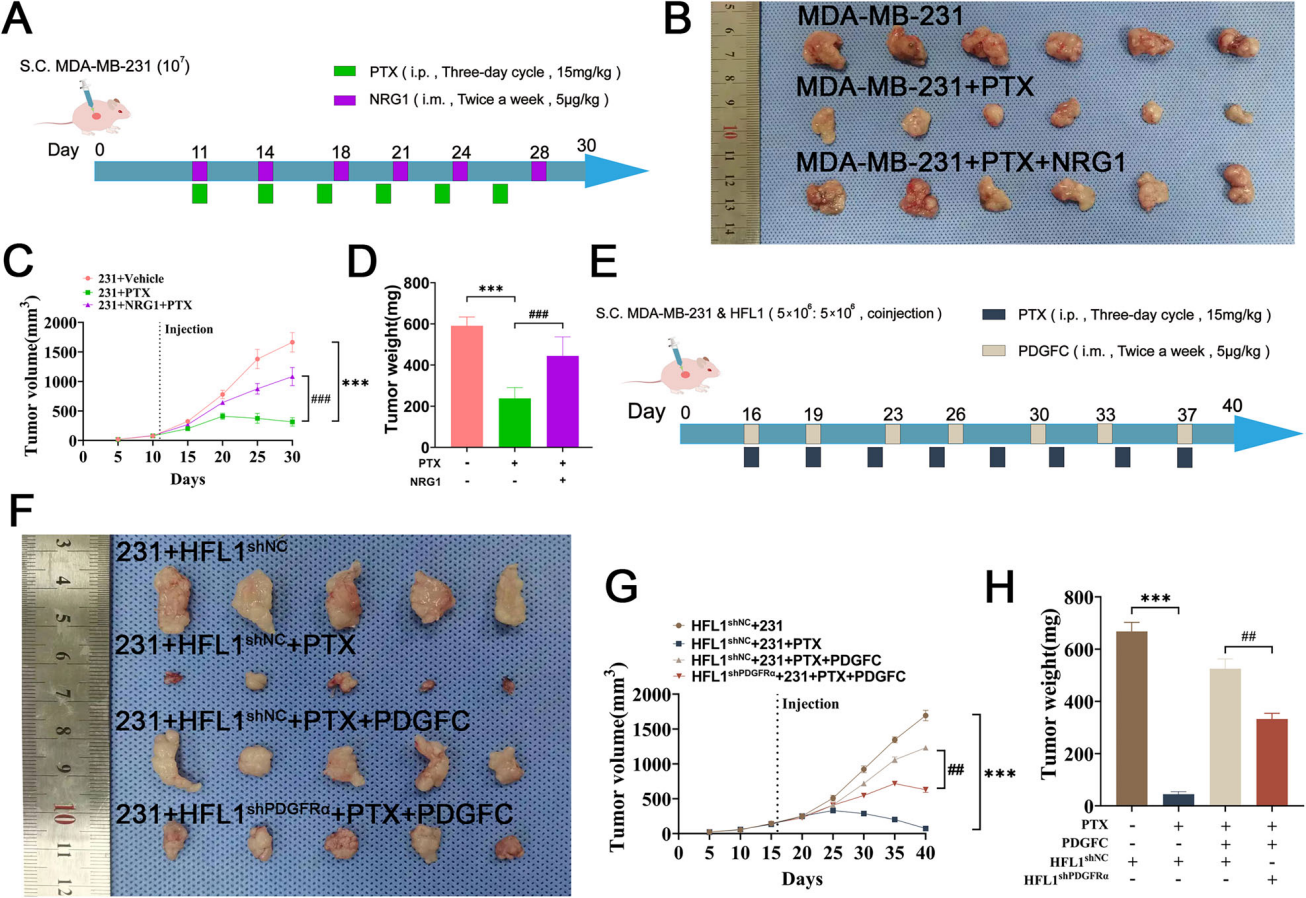
NRG1 and PDGFC promote PTX resistance in BC in vivo. **A** Schematic diagram of the mouse BC xenograft model established using MDA-MB-231 cells. PTX (15 mg/kg, intraperitoneally, every three days) and recombinant NRG1 protein (5 μg/kg, intratumorally, twice a week) were administered starting on day 11 post-tumor implantation. **B** Representative images of excised tumors from the mice (*n* = 6). **C** Tumor volumes were measured every five days, and mice were sacrificed on day 30 for analysis (*n* = 6). **D** Quantification of the excised tumor weights (*n* = 6). **E** MDA-MB-231 cells and HFL1 cells were co-injected at a 1:1 ratio into the subcutaneous tissue of nude mice to establish a BC model. PTX (15 mg/kg, intraperitoneally, every three days) and recombinant PDGFC protein (5 μg/kg, intratumorally, twice a week) were administered starting on day 16 post-tumor implantation. **F** Representative images of excised tumors (*n* = 6). **G** Quantification of tumor volume changes in mice (*n* = 6). **H** Quantification of excised tumor weights (*n* = 6). Data are expressed as the mean ± S.D. for all panels: ^**##**^*P* < 0.01, ***^, **###**^*P* < 0.001.

To further validate the roles of NRG1 and PDGFC in promoting PTX resistance in BC in vivo, we established ectopic tumor models using nude mice. After constructing a BC model with MDA-MB-231 cells, PTX was administered intraperitoneally, and NRG1 was injected intratumorally to evaluate its effect on PTX resistance (Fig. 6A). The results showed that intratumoral injection of NRG1 significantly increased tumor volume and weight after chemotherapy (Fig. 6B–D). To investigate the impact of PDGFC-mediated fibroblast activation on PTX resistance, PDGFRα was knocked down in human embryonic lung fibroblasts (HFL1) using lentiviral transfection (Fig. S5A, B). Consistent with primary fibroblast experiments, exogenous PDGFC significantly promoted FAP expression as well as NRG1 expression and secretion in HFL1, while PDGFRα knockdown inhibited these effects (Fig. S5C, D). Co-injection of MDA-MB-231 cells and HFL1 cells at a 1:1 ratio into the subcutaneous tissue of nude mice was followed by intraperitoneal administration of PTX and intratumoral injection of PDGFC to assess its impact on PTX resistance (Fig. 6E). The results indicated that exogenous PDGFC significantly enhanced PTX resistance in vivo, while PDGFRα knockdown in HFL1 attenuated the promotive effect of PDGFC on PTX resistance (Fig. 6F–H). These findings suggest that NRG1 promotes PTX resistance in BC cells in vivo, and PDGFC-mediated fibroblast activation further enhances PTX resistance.

## Discussion

Despite significant advancements in BC treatment in recent years, chemoresistance remains a major challenge in its clinical management [31]. Increasing evidence indicates that CAFs within the TME play a critical role in regulating chemoresistance [7, 32]. However, the molecular interactions between CAFs and tumor cells that drive chemoresistance have yet to be fully elucidated. In this study, we established a co-culture system and observed that CAFs significantly promoted PTX resistance in BC cells. Based on existing literature, CAF-derived NRG1 has been shown to enhance anti-androgen resistance in prostate cancer and trastuzumab resistance in BC [15, 17]. Building on these findings, we further validated the potential role of CAF-derived NRG1 in mediating PTX resistance in BC.

Neuregulins (NRGs) are members of the epidermal growth factor (EGF) protein family, mediating intercellular signaling and predominantly expressed in tissues such as the brain and breast [33, 34]. NRGs are encoded by four genes (NRG1-4), and NRG1 contains two types of EGF-like domains: α and β [35]. The primary receptors of NRG1 are ErbBs, which are classical members of the human epidermal growth factor receptor (HER) family [36]. The EGF-like domain of NRG1 promotes conformational changes in ErbBs, leading to their dimerization and activation, which subsequently results in phosphorylation of the downstream signaling molecule AKT [26]. In this study, we demonstrated that CAF-secreted NRG1 activates the AKT/mTOR signaling pathway in BC cells, thereby significantly enhancing their resistance to PTX.

Ferroptosis is a newly identified form of PCD, distinct from other types of cell death [37]. The occurrence of ferroptosis is dependent on intracellular iron overload and the accumulation of lipid peroxides [37]. During chemotherapy of tumor cells, ferroptosis often occurs in conjunction with tumor cell death [38]. Numerous studies have shown that promoting ferroptosis in cancer cells can enhance the efficacy of chemotherapy in killing tumor cells [38, 39]. The GSH-GPX4 system is the most extensively studied ferroptosis resistance pathway, playing a crucial role in cellular defense against ferroptosis [40]. The solute carrier family 7 member 11 (SLC7A11) is an upstream regulator of the GSH-GPX4 system, and as a membrane protein, its main function is to transport glutamate and cysteine across the cell membrane. Cysteine serves as the raw material for GSH synthesis, thereby exerting antioxidant effects [41]. In this study, we found that NRG1, by activating the AKT/mTOR signaling pathway, significantly enhances the signaling of the SLC7A11-GSH-GPX4 axis in BC cells, thereby substantially increasing the ability of BC cells to escape ferroptosis under PTX stress.

Platelet-derived growth factor C (PDGFC) is an important member of the PDGF family, consisting of a dimer formed by two polypeptide chains [42]. PDGFC plays a crucial role in the pathophysiology of various diseases, including angiogenesis and repair, fibrosis, tumorigenesis, progression, and metastasis [21–23]. Studies have reported that PDGFC derived from cancer cells is vital for the activation of fibroblasts, which promotes the lung colonization of BC cells [23]. In this study, we observed that BC cells express PDGFC at higher levels compared to normal breast epithelial cells, and exogenous PDGFC significantly enhances fibroblast activation and the expression and secretion of NRG1. Additionally, exogenous NRG1 significantly increases PDGFC expression in BC cells. These findings suggest that NRG1 derived from CAFs and PDGFC from BC cells may form a positive feedback loop, further driving the vicious cycle of PTX resistance in BC cells. However, the underlying molecular mechanisms by which PDGFC promotes fibroblast activation and high NRG1 expression still require further investigation. Meanwhile, we observed differential expression of PDGFC between different cell lines, such as MCF-7 and MDA-MB-231, which may be attributed to the distinct breast cancer subtypes or other tumor cell heterogeneity. Comparing PDGFC expression across a broader range of breast cancer molecular subtypes, as well as evaluating the activity of the PDGFC/NRG1 signaling axis between tumor cells and fibroblasts, will provide valuable insights for the development of more precise therapeutic strategies in breast cancer.

In our study, consistent with the cellular level, exogenous NRG1 and PDGFC stimulation of fibroblasts both contributed to the development of PTX resistance in BC cells in vivo. These in vitro and in vivo experimental results indicate that NRG1 and PDGFC in the TME significantly promote chemoresistance in BC, suggesting that chemotherapy combined with targeted blockade of the NRG1/PDGFC signaling between CAFs and cancer cells may be an effective strategy for improving BC treatment.

## Conclusion

**Fig. 7:**
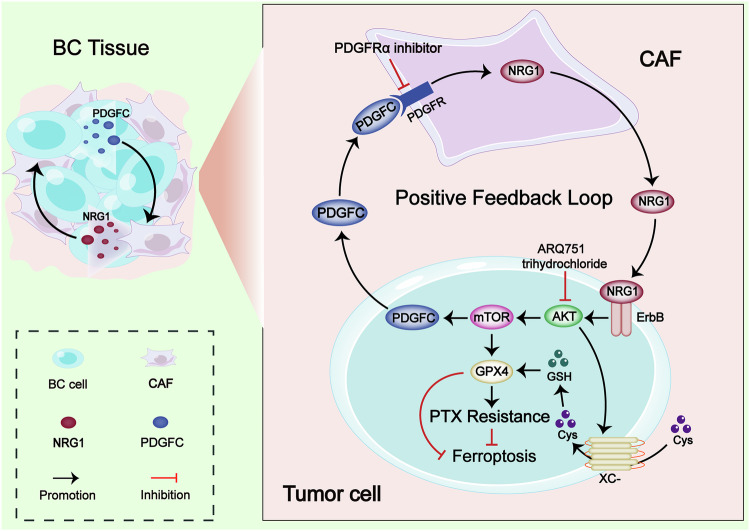
The NRG1/PDGFC positive feedback loop between CAFs and BC cells promotes PTX resistance in BC. NRG1 derived from CAFs promotes ferroptosis escape in BC cells through the activation of the AKT/mTOR signaling pathway, thereby promoting PTX resistance in BC cells. Meanwhile, activation of the AKT/mTOR signaling pathway induces high expression of PDGFC, and BC cells release PDGFC to activate fibroblasts and further release NRG1, ultimately forming the NRG1/PDGFC positive feedback loop.

In this study, we revealed that NRG1 derived from CAFs in the BC microenvironment promotes PTX resistance in BC cells by activating the AKT/mTOR signaling pathway, which facilitates the escape from ferroptosis under PTX pressure. Furthermore, the activation of AKT/mTOR signaling by NRG1 induces high expression of PDGFC in cancer cells, which in turn promotes the activation of fibroblasts and NRG1 expression through the release of PDGFC, forming a positive feedback loop between NRG1 and PDGFC. This mechanism promotes the malignant cycle of PTX resistance in BC cells (Fig. 7). These findings highlight the potential opportunity of targeting the NRG1/PDGFC signaling pathway to limit chemoresistance in BC.

## Materials and methods

### Primary fibroblast isolation

BC tissues and paired adjacent tissues were obtained from the Affiliated Hospital of Shandong Second Medical University, with pathological diagnoses confirming invasive ductal carcinoma. CAFs were extracted from the BC tissues, and NFs were derived from adjacent normal tissues using the tissue block method. Fresh BC tissues were first washed with precooled PBS buffer containing penicillin-streptomycin solution. Fat, blood vessels, and necrotic tissues were removed with scissors, and the remaining tissues were cut into approximately 1 mm³ pieces (tissues located 5 cm away from the tumor were considered adjacent normal tissues). The tissue fragments were then placed at the bottom of a culture dish and covered with fetal bovine serum (FBS) containing penicillin-streptomycin. The culture dish was incubated in a cell culture incubator for 6 h. Once the tissue fragments adhered to the dish, 10% FBS and 1% penicillin-streptomycin-supplemented DMEM medium was carefully added, and the cells were cultured for 1–2 weeks. When cells began migrating from the edges of the tissue under a microscope, they were digested with trypsin and passaged for subsequent experiments. The collection of human samples was approved by the Ethics Committee of Shandong Second Medical University (Approval No.: 2023YX069).

### Cell culture

The MDA-MB-231, MCF-7, MCF-10A, and HFL1 cell lines were preserved long-term by our research group. All cell lines were purchased from the American Type Culture Collection (ATCC) and authenticated by STR profiling. MDA-MB-231, MCF-7, and MCF-10A cells were cultured in DMEM medium supplemented with 10% FBS and 1% penicillin-streptomycin, while HFL1 cells were cultured in Ham’s F-12 medium containing 10% FBS and 1% penicillin-streptomycin. All cells were maintained in a 37 °C incubator with 5% CO₂. When the cells reached 80%-90% confluency, they were passaged for further culture.

### Co-culture system

When CAFs and NFs were in the logarithmic growth phase, the culture medium was replaced with serum-free DMEM and incubated for 24 h. The supernatant was then collected, centrifuged, and filtered, followed by supplementation with 10% FBS to prepare a complete medium. This prepared medium was added to BC cells for co-culture. The co-culture system of BC cells and fibroblasts was established using the same procedure described above.

### Drug treatment

PDGFC recombinant protein (HY-P7279), Paclitaxel (PTX, HY-B0015), ARQ 751 trihydrochloride (HY-137458A), Erastin (HY-15763), Nigericin (HY-127019), Hydroxyurea (HY-B0313), Wogonoside (HY-N0399), and PDGFRα kinase inhibitor 1 (HY-111507) were purchased from MedChemExpress (USA), while NRG1 recombinant protein was obtained from Cell Signaling Technology (26941). To investigate the effect of CAFs on BC drug resistance, BC cells were co-cultured with supernatants from CAFs and NFs for 24 h, followed by the addition of PTX (20 nM) and incubation for another 24 h. To explore the role of NRG1 in BC cell drug resistance, PTX (20 nM) and ARQ 751 trihydrochloride (10 nM) were first added and incubated for 30 min, then supplemented with NRG1 recombinant protein (50 ng/mL) for 2 days. In studying the effect of NRG1 on PCD in BC cells, the cells were treated with Erastin (5 μM), Nigericin (2 μg/mL), Hydroxyurea (2 mM), or Wogonoside (100 μM) for 30 min, followed by exogenous supplementation of NRG1 recombinant protein (50 ng/mL) and incubation for 2 days. For the investigation of PDGFC’s effect on fibroblasts, fibroblasts at appropriate densities were treated with exogenous PDGFC recombinant protein (50 ng/mL) and incubated for 2 days. To evaluate the impact of PDGFRα inhibition on the activation of fibroblasts by cancer cells, PDGFRα kinase inhibitor 1 (0.5 μM) was added for 30 min, followed by co-culture with BC cell supernatant for 2 days.

### CCK-8 assay

Logarithmically growing cancer cells were seeded into 96-well plates at an appropriate density. After drug treatment, recombinant protein administration, or co-culture, 10 μL of CCK-8 solution was added to each well and incubated at 37 °C for 2–4 h. The absorbance at 450 nm was measured using a microplate reader, and the cell viability of each group was calculated accordingly.

### siRNA transfection

NRG1 siRNA was purchased from Shanghai GenePharma Co., Ltd. The siRNA sequence (5’ to 3’) is shown in Table 1. Cells were seeded into 6-well plates and, when the cell density reached approximately 70%, the culture medium was replaced with OPTI-MEM serum-free medium, and incubated for 30 min. The transfection reagent GP-transfect-Mate was mixed with an appropriate amount of siRNA to form a transfection complex and incubated at room temperature for 20 min. The transfection complex was then added to the cells and incubated in the culture incubator for 8 h. After 8 h of transfection, the transfection efficiency was observed under a fluorescence microscope, and the medium was replaced with DMEM containing 10% FBS. Cells were further cultured for 48 h before being collected for subsequent experiments.

**Table 1:** Gene sequence of siNRG1 (5’ to 3’).

siRNA	Sequence (5’ to 3’)
Negative control	Sense: UUCUCCGAACGUGUCACGUTT
	Antisense: ACGUGACACGUUCGGAGAATT
siNRG1-1	Sense: GGGAAUGAAUUGAAUCGAATT
	Antisense: UUCGAUUCAAUUCAUUCCCTT
siNRG1-2	Sense: GGCUGAUUCUGGAGAGUAUTT
	Antisense: AUACUCUCCAGAAUCAGCCTT
siNRG1-3	Sense: GUGGAAUCAAACGAGAUCATT
	Antisense: UGAUCUCGUUUGAUUCCACTT
siNRG1-4	Sense: GGAGCAAAUACUUCUUCAUTT
	Antisense: AUGAAGAAGUAUUUGCUCCTT

### Western blot

Total proteins were extracted from cells using pre-chilled lysis buffer containing protease inhibitors. After extraction, protein concentrations were determined using a BCA protein assay kit (CW014, Kangwei, China), and protein concentrations were normalized across different groups. Following SDS-PAGE, proteins were transferred to a PVDF membrane. The membrane was blocked with 5% non-fat milk for 1 h. The membrane was then incubated overnight at 4 °C with a solution containing the primary antibody specific to the target protein. The next day, the membrane was washed three times with TBST buffer and incubated with the secondary antibody solution at room temperature for 1 h. After washing, the membrane was developed using ECL chemiluminescent solution and exposed. The details of the primary and secondary antibodies used in our experiments are listed in Table 2.

**Table 2:** The antibody information used in this study.

Antibody name	Dilution	Catalogue number and supplier
*Western blot*		
alpha SMA Antibody	1:1000	BF9212, Affinity
FAP (E1V9V) Rabbit mAb	1:1000	66562, Cell Signaling Technology
FSP1/S100A4 Monoclonal antibody	1:1000	66489-1-Ig, Proteintech
Heregulin Antibody	1:1000	2573, Cell Signaling Technology
GAPDH Monoclonal antibody	1:3000	60004-1-Ig, Proteintech
AKT Monoclonal antibody	1:2000	60203-2-Ig, Proteintech
Phospho-AKT (Ser473) Monoclonal antibody	1:2000	66444-1-Ig, Proteintech
mTOR Monoclonal antibody	1:2000	66888-1-Ig, Proteintech
Phospho-mTOR (Ser2448) Monoclonal antibody	1:2000	67778-1-Ig, Proteintech
SLC7A11/xCT Polyclonal antibody	1:2000	26864-1-AP, Proteintech
GPX4 Monoclonal antibody	1:3000	67763-1-Ig, Proteintech
Bcl2 Monoclonal antibody	1:2000	68103-1-Ig, Proteintech
BAX Monoclonal antibody	1:3000	60267-1-Ig, Proteintech
Caspase 3/P17/P19 Polyclonal antibody	1:1000	19677-1-AP, Proteintech
PDGFC Polyclonal antibody	1:1000	55076-1-AP, Proteintech
PDGFR alpha/CD140a Recombinant antibody	1:1000	84383-2-RR, Proteintech
HRP-conjugated Goat Anti-Rabbit IgG(H + L)	1:3000	SA00001-2, Proteintech
HRP-conjugated Goat Anti-Mouse IgG(H + L)	1:3000	SA00001-1, Proteintech
*Immunofluorescence*		
alpha SMA Antibody	1:200	BF9212, Affinity
FAP (E1V9V) Rabbit mAb	1:200	66562, Cell Signaling Technology
FSP1/S100A4 Monoclonal antibody	1:200	66489-1-Ig, Proteintech
CoraLite488-conjugated goat anti-mouse IgG	1:200	SA00013-1, Proteintech
CoraLite594 – conjugated Goat Anti-Mouse IgG	1:200	SA00013-3, Proteintech
Goat Anti-Rabbit IgG H&L (Alexa Fluor® 594)	1:200	ab150080, Abcam
Goat Anti-Rabbit IgG H&L (Alexa Fluor® 488)	1:200	ab150077, Abcam

### Extraction of secreted proteins from culture supernatant

Secreted proteins from the cell culture supernatant were extracted using the methanol-chloroform method. First, the culture supernatant was collected and centrifuged at 3000 rpm for 5 min to remove cell debris. The supernatant was then mixed with an equal volume of methanol and one-quarter volume of chloroform, and thoroughly vortexed. The mixture was centrifuged at 13,000 rpm for 5 min, resulting in three layers. The upper and lower layers were discarded, and the white middle layer was collected. To the white material, 1 mL of methanol was added, mixed well, and centrifuged at 12,000 rpm for 10 min. The methanol was discarded, and an appropriate amount of lysis buffer was added, followed by thorough mixing. Finally, loading buffer was added, and the sample was boiled for 10 min. The cell lysates and secreted proteins were quantified using the BCA protein assay, and the concentration of the secreted proteins was adjusted according to the ratio.

### Immunofluorescence

Cells were seeded onto sterile glass coverslips in 24-well plates, and 500 μL complete culture medium was added for incubation in the culture chamber. Once cells reached an appropriate density, they were washed three times with PBS, followed by fixation with 4% paraformaldehyde for 15 min. Then, 500 μL of 1% Triton X-100 was added to each well, and the cells were incubated at room temperature for 15 min to permeabilize the cell membrane. After washing with PBS, goat serum was added for blocking at room temperature for 1 h. The goat serum was removed, and 200 μL of primary antibody solution was added, diluted at the following ratios: α-SMA (1:200), FAP (1:200), S100A4 (1:200), and incubated overnight at 4 °C. The next day, the slides were washed with PBS and incubated with secondary fluorescent antibody (dilution ratio 1:200) at room temperature for 1 h. DAPI staining solution was added to each well, and the cells were incubated in the dark for 5 min. Afterward, the glass coverslips were mounted with 10 μL of anti-fade reagent. Finally, images were captured using a laser scanning confocal microscope.

### Enzyme-linked immunosorbent assay (ELISA)

Human Neuregulin 1 (NRG1) ELISA Kit (JL15126, JONLNBIO) and Human Platelet-Derived Growth Factor C (PDGF-C) ELISA Kit (JL23863, JONLNBIO) were used to measure the concentrations of NRG1 and PDGFC in the fibroblast and BC cell culture supernatants, respectively. The experimental procedure followed the instructions provided by the kit manufacturers. After centrifugation and filtration of the cell supernatants, ELISA was performed. Upon completion of the reaction, OD values were measured at 450 nm, and the concentrations were calculated based on the standard curve.

### Colony-formation assay

After preparing a single-cell suspension of cancer cells, 500 cells per well were seeded into 6-well plates. The complete medium or culture supernatant containing PTX and recombinant protein was replaced every 3 days. After 15 days of continuous culture, the cells were fixed with 4% paraformaldehyde. Subsequently, the cells were stained with Giemsa stain. Finally, the number of colonies with a diameter greater than 50 μm was counted.

### Transmission electron microscopy (TEM)

Cell samples treated with interventions were collected and fixed in 2.5% glutaraldehyde at 4 °C for 4 h. After dehydration and embedding, ultrathin sections were prepared using an ultramicrotome. The sections were stained with heavy metals and then observed under a transmission electron microscope.

### MDA content measurement

Malondialdehyde (MDA) is the main end product of lipid peroxidation in cells and serves as a good indicator of ferroptosis levels. MDA levels in BC cells were measured using the MDA Content Detection Kit (BC0025, Solarbio, Beijing, China). The experiment was conducted according to the manufacturer’s instructions.

### GSH/GSSG content measurement

Glutathione (GSH) is an important antioxidant in cells, while glutathione disulfide (GSSG) is the oxidized form of GSH. By measuring the GSH/GSSG ratio, the antioxidant capacity of cells can be assessed, which in turn reflects the level of ferroptosis. Total GSH content and the GSH/GSSG ratio in BC cells were measured using the GSH and GSSG Detection Kit (S0053, Beyotime Biotechnology, China).

### JC-1 assay

The JC-1 Mitochondrial Membrane Potential Assay Kit (C2006, Biotime Biotechnology Co., Ltd.) was used to detect changes in the mitochondrial membrane potential of BC cells after drug treatment. When the mitochondrial membrane potential is low, JC-1 does not aggregate in the mitochondrial matrix, remaining in its monomeric form and emitting green fluorescence. When the mitochondrial membrane potential is high, JC-1 aggregates in the mitochondrial matrix, forming a polymer that emits red fluorescence. The experimental procedure was carried out according to the instructions provided by the kit.

### Cell line-derived xenograft (CDX)

Female BALB/c nude mice aged 6 to 8 weeks were purchased from Jinan Pengyue Laboratory Animal Breeding Co., Ltd., and housed in SPF-grade individually ventilated cages with temperature maintained at 20–25 °C, humidity at 50–60%, and provided with adequate food and water. For studying the in vivo effect of NRG1 on chemoresistance in BC, the mice were randomly divided into three groups: MDA-MB-231 + Vehicle, MDA-MB-231 + PTX, and MDA-MB-231 + PTX + NRG1, with 6 mice per group. Each mouse was subcutaneously injected with 1 × 10⁷ MDA-MB-231 cells in good growth condition. Tumor size was measured every 3 days using calipers. When the tumor volume reached approximately 100 mm³, PTX (15 mg/kg) was administered intraperitoneally every 3 days, and NRG1 (5 μg/kg) was injected intratumorally twice a week. Tumor size was recorded every 3 days, and after 30 days, the mice were euthanized to collect tumor samples for imaging and weighing. To study the effect of PDGFC-stimulated fibroblasts on chemoresistance in BC cells, the mice were randomly divided into four groups: MDA-MB-231 + HFL1^shNC^, MDA-MB-231 + HFL1^shNC^ + PTX, MDA-MB-231 + HFL1^shNC^ + PTX + PDGFC, and MDA-MB-231 + HFL1^shPDGFRα^ + PTX + PDGFC. HFL1 cells transfected with fluorescent protein-labeled lentivirus shRNA-PDGFRα or shRNA-NC were mixed with MDA-MB-231 cells at a 1:1 ratio (5 × 10⁶:5 × 10⁶ cells) and subcutaneously injected into the nude mice. When the tumor volume reached approximately 100 mm³, PTX (15 mg/kg) was administered intraperitoneally every 3 days, and PDGFC (5 μg/kg) was injected intratumorally twice a week. Tumor size was recorded every 3 days, and after 40 days, the mice were euthanized to collect tumor samples for imaging and weighing. All animal experiments were performed in accordance with National Institutes of Health guidelines and were approved by the Ethics Committee of Shandong Second Medical University (Approval Number: 2023SDL184).

### Statistical analysis

All statistical analyses were performed using GraphPad Prism version 9.0 (GraphPad Software, San Diego, CA, USA). Data are presented as mean ± standard deviation (s.d.) unless otherwise specified. The sample size (*n*) for each experimental group is indicated in the figure legends and represents independent biological replicates. For in vitro experiments, each assay was independently repeated at least three times using different biological samples or passages. For animal experiments, each group contained at least six animals as indicated in the figure legends. No formal statistical methods were used to predetermine sample size. Sample sizes were based on prior publications in the field and established laboratory experience with similar models.

No animals or samples were excluded from the analysis unless they were technically flawed. No formal randomization method was used to assign samples or animals in this study; however, group allocation was conducted in a non-biased manner to ensure that the initial conditions of the samples or animals were as comparable as possible. No formal blinding was implemented during the experiments or outcome assessments. However, data analysis was performed by researchers not directly involved in the treatment administration to minimize potential bias.

All statistical tests used in this study were selected based on the data distribution and experimental design. Normality of data was assessed using the Shapiro-Wilk test. For normally distributed data, parametric tests such as unpaired two-sided Student’s t-tests or ANOVA were applied. Comparisons between two groups were made using unpaired two-tailed Student’s t-tests. For comparisons involving more than two groups, one-way analysis of variance (ANOVA). *P* < 0.05 were considered statistically significant. All center values reflect the mean, and error bars indicate s.d. unless otherwise noted.

## Supplementary information

Supplementary Figures

Original data

## Data Availability

The data supporting the findings of this study are available from the corresponding author upon reasonable request.
