# Supplementary material for: NRG1/PDGFC loop between fibroblasts and cancer cells drives paclitaxel resistance via ferroptosis suppression in breast cancer
Source: Cell Death Discov. 2025 Nov 10;11:520. doi: 10.1038/s41420-025-02785-2 (PMC12603068; doi:10.1038/s41420-025-02785-2)
Supplement: Supplementary file 1 — Supplementary Figures [file 41420_2025_2785_MOESM1_ESM.docx]

**Supplementary Figures**

**Figure S1.**

**
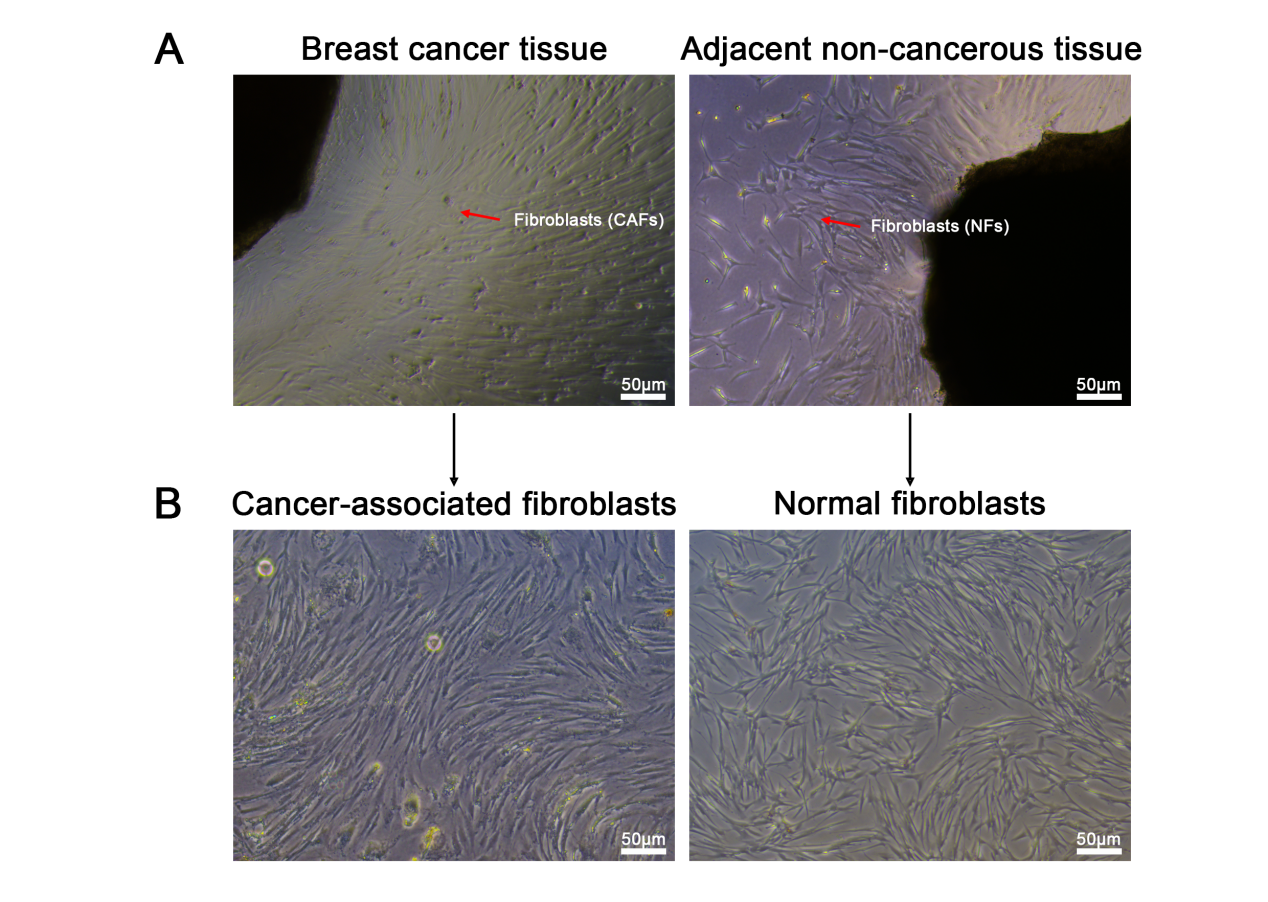
**

**Figure S1. Primary isolation of cancer-associated fibroblasts (CAFs) and normal fibroblasts (NFs).** (A) Microscopic images of primary CAFs and NFs isolated from breast cancer tissues and adjacent normal tissues using the tissue explant method. Scale bar = 50 μm. (B) Microscopic images showing CAFs and NFs in the logarithmic growth phase after subculture. Scale bar = 50 μm.

**Figure S2.**

**
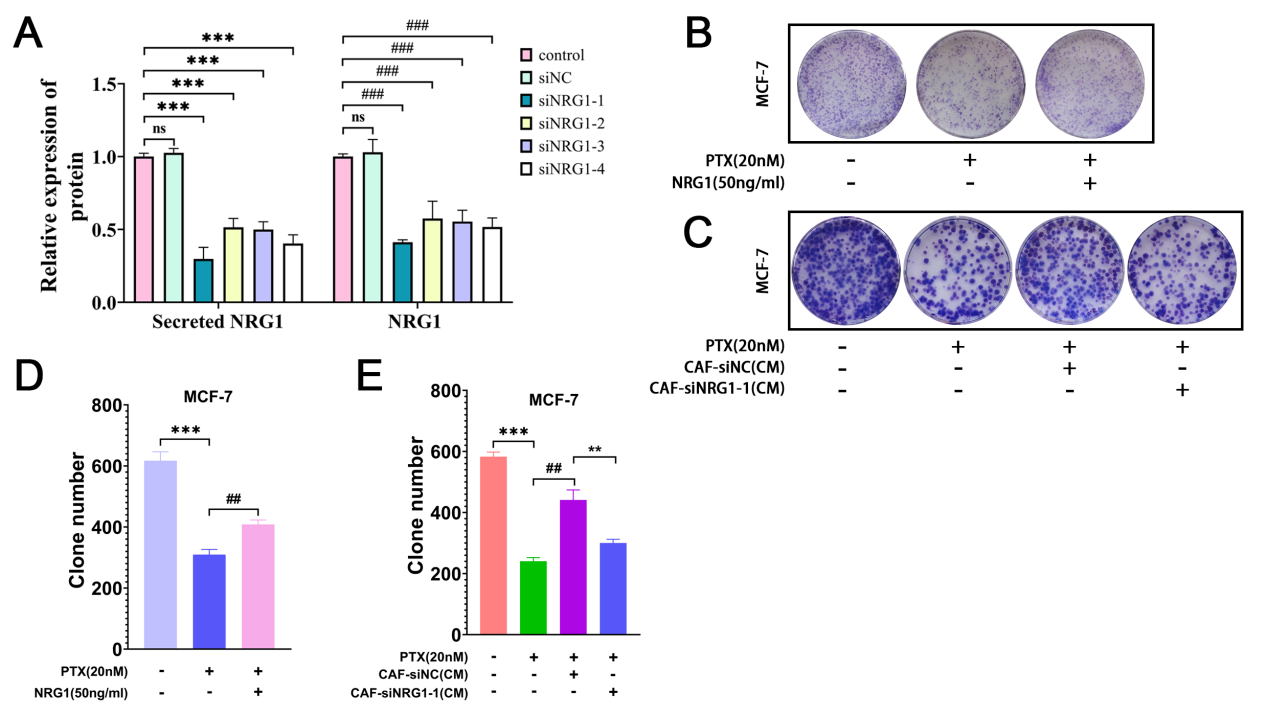
**

**Figure S2. The promotion of paclitaxel resistance in breast cancer cells by CAF-derived NRG1.** (A) Quantification of the Western blot results from Figure 2F. (B-D) Colony formation assays showing the effects of exogenous NRG1 and NRG1 knockdown in CAFs on the colony-forming ability of MCF-7 cells. Data are expressed as the mean ± S.D. for all panels: ns = no significance, **^##,^ ****P < 0.01, ^✱✱✱,^ **^###^**P < 0.001.

**Figure S3.**

**
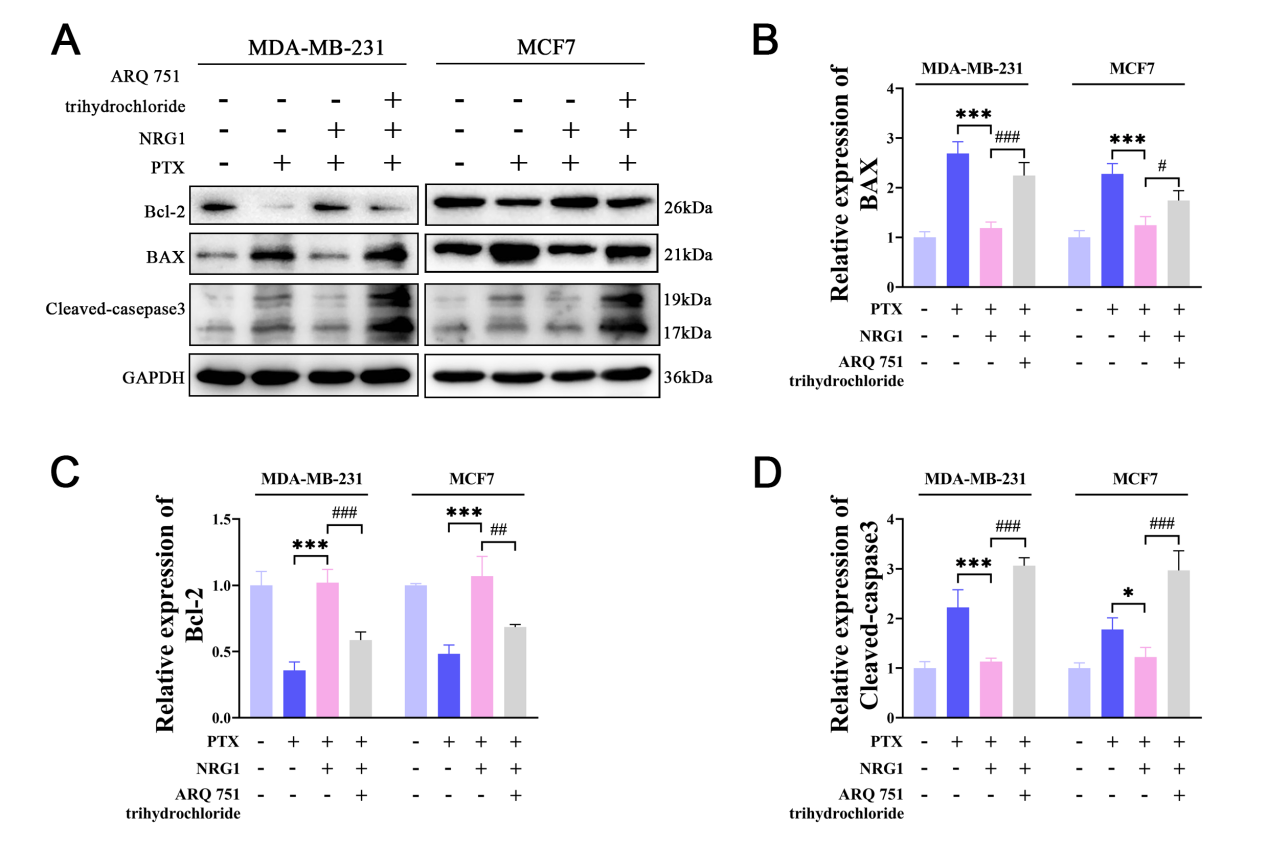
**

**Figure S3. Exogenous NRG1 exerts an inhibitory effect on PTX-induced apoptosis.** (A-D) Western blot showed the effects of exogenous NRG1 and an AKT inhibitor on the expression levels of BAX, Bcl-2, and Cleaved-caspase3 in breast cancer cells. Data are expressed as the mean ± S.D. for all panels: ^✱,^ **^#^**P < 0.05, **^##^**P < 0.01, ^✱✱✱,^ **^###^**P < 0.001.

**Figure S4.**

**
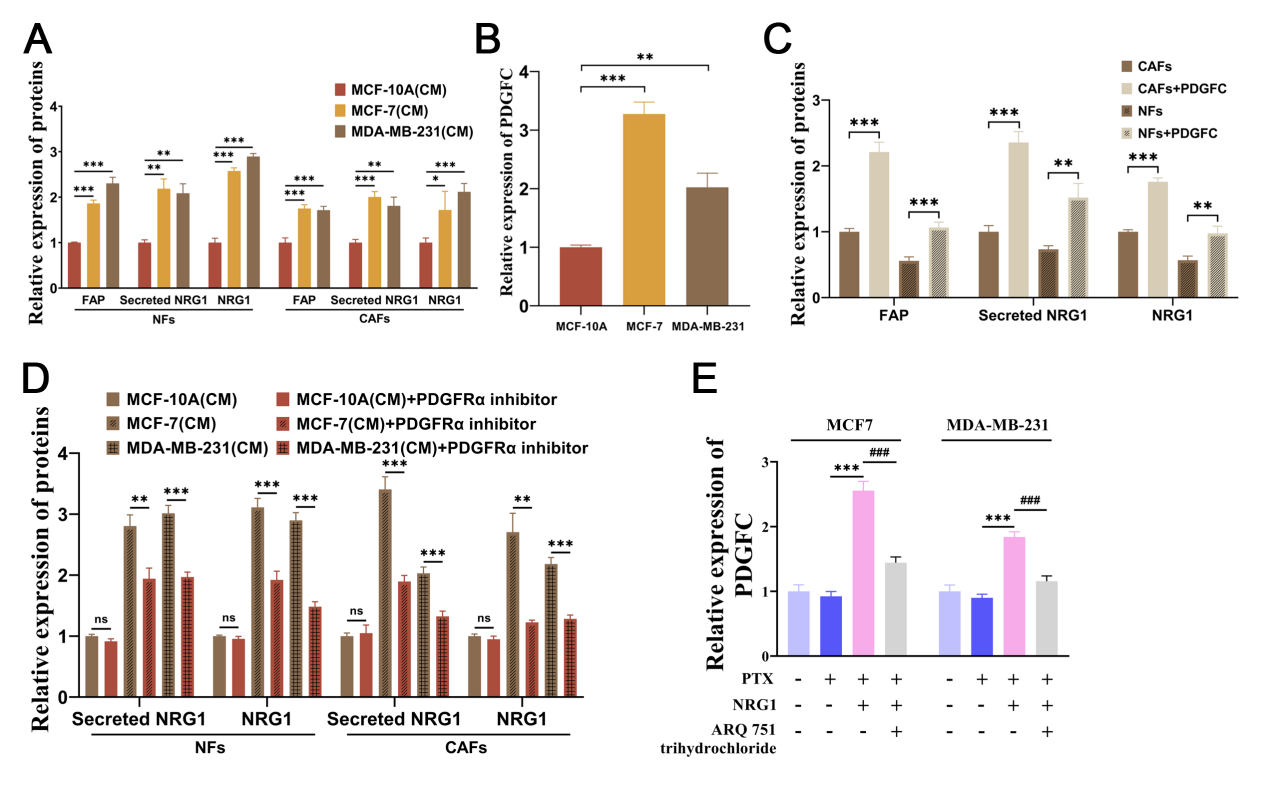
**

**Figure S4. PDGFC released by BC cells activates fibroblasts and promotes their high expression of NRG1.** (A-E) Quantification of the Western blot results shown in Figure 5A, 5B, 5E, 5F, and 5G. Data are expressed as the mean ± S.D. for all panels: ns = no significance, ^✱^P < 0.05, ^✱✱^P < 0.01, ^✱✱✱,^ **^###^**P < 0.001.

**Figure S5.**

**
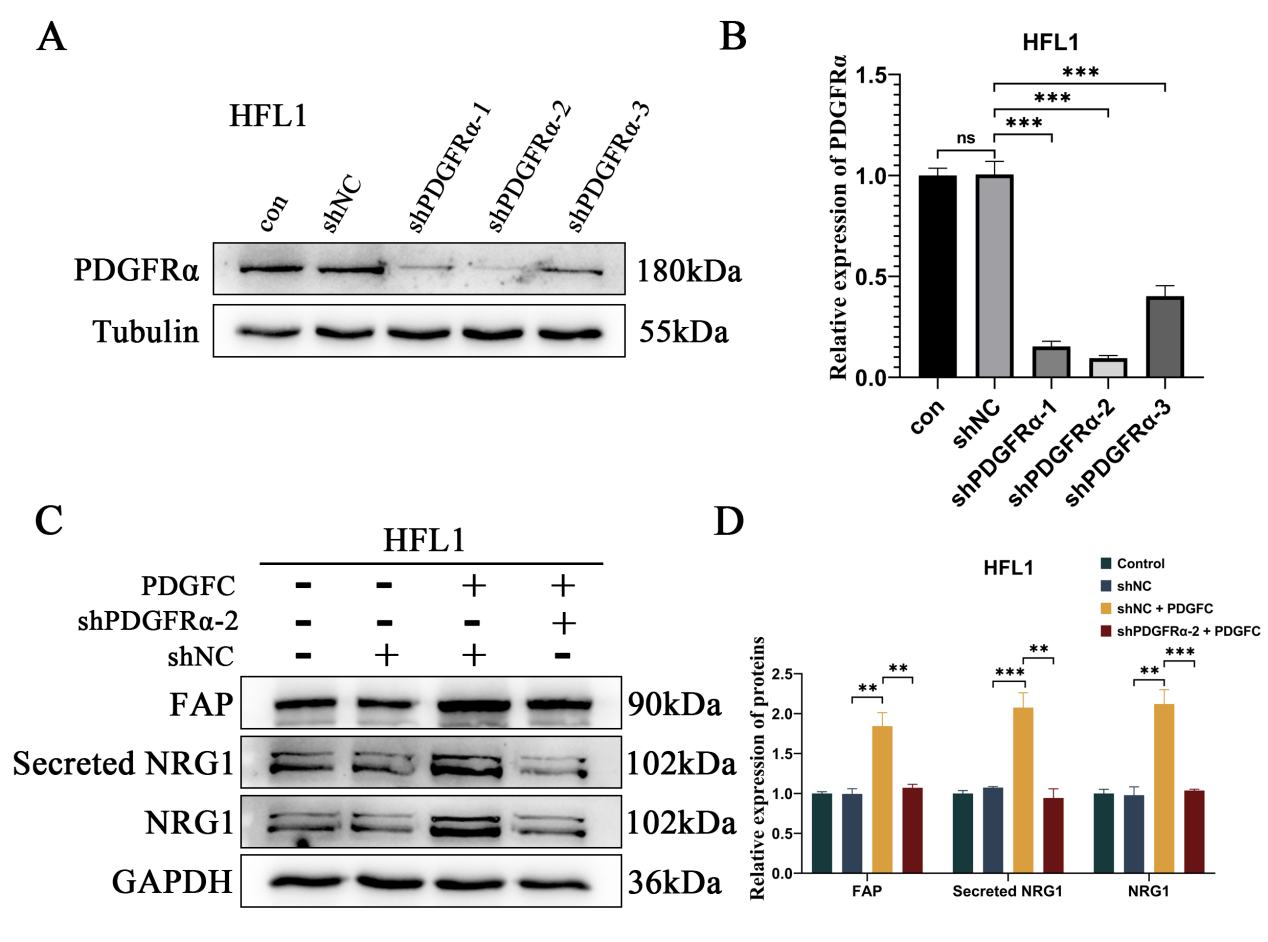
**

**Figure S5. The effects of stable PDGFRα knockdown and exogenous PDGFC on HFL1 cells.** (A-B) Stable PDGFRα knockdown in HFL1 cells was achieved using lentivirus, and the knockdown efficiency was observed by Western blot. (C-D) Western blot analysis showed the effects of exogenous PDGFC and stable PDGFRα knockdown on the expression FAP and the expression and secretion of NRG1 in HFL1 cells. Data are expressed as the mean ± S.D. for all panels: ns = no significance, ^✱✱^P < 0.01, ^✱✱✱^P < 0.001.
