# Supplementary figures and images for: NRG1/PDGFC loop between fibroblasts and cancer cells drives paclitaxel resistance via ferroptosis suppression in breast cancer
Source: Cell Death Discov. 2025 Nov 10;11:520. doi: 10.1038/s41420-025-02785-2 (PMC12603068; doi:10.1038/s41420-025-02785-2)

# Original data

Fig 1B

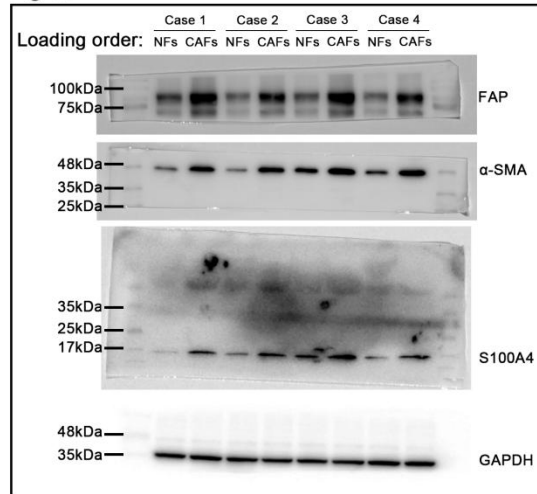

Fig 2A

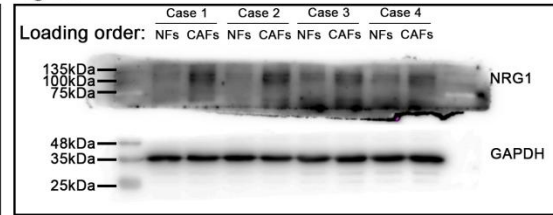

Fig 2C

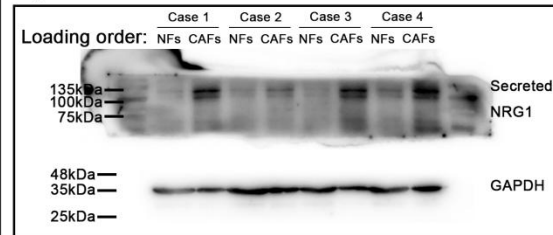

Fig 2F

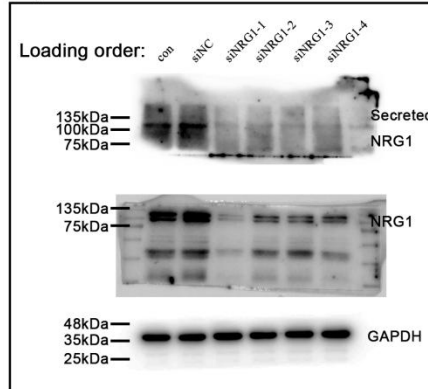

Fig 3D

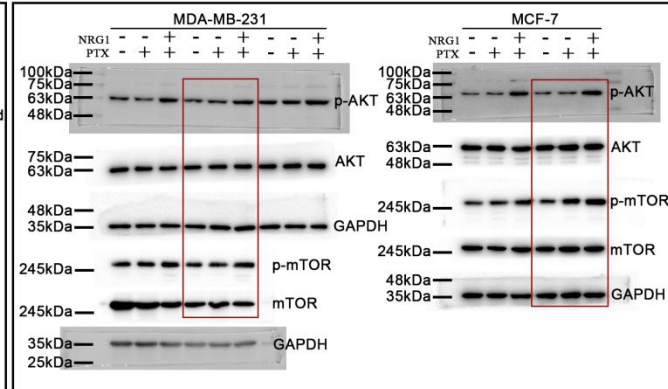

Fig 3F

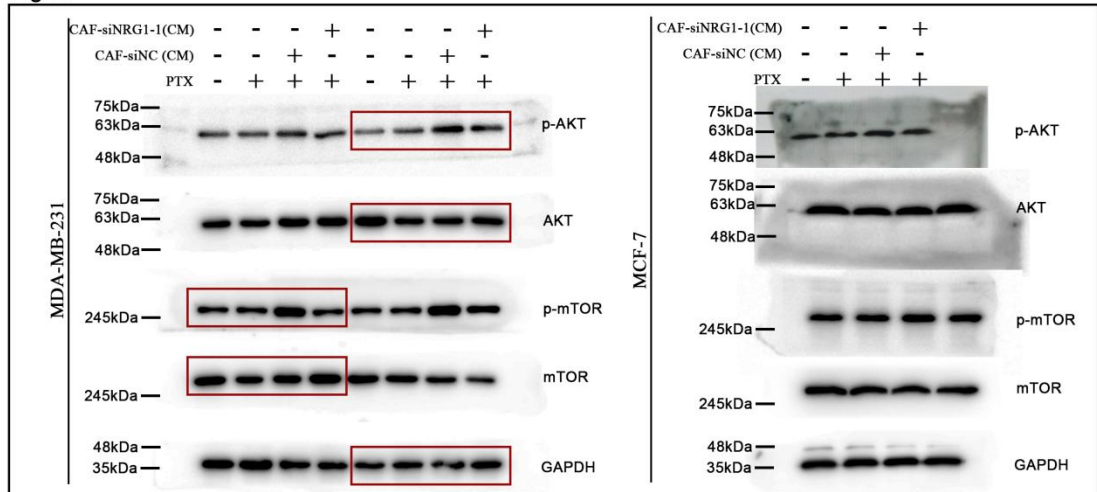

Fig 4A

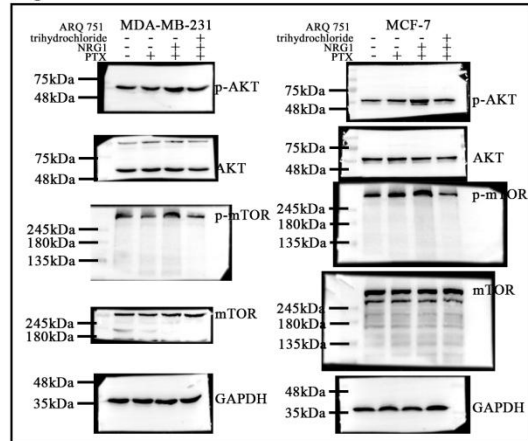

Fig 4C

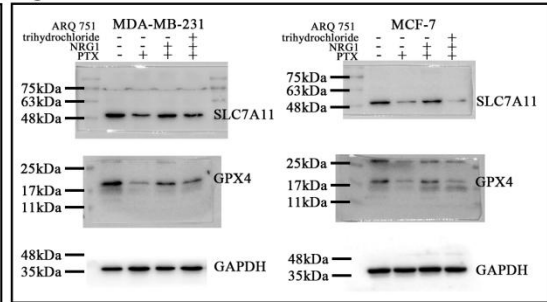

Fig 5A

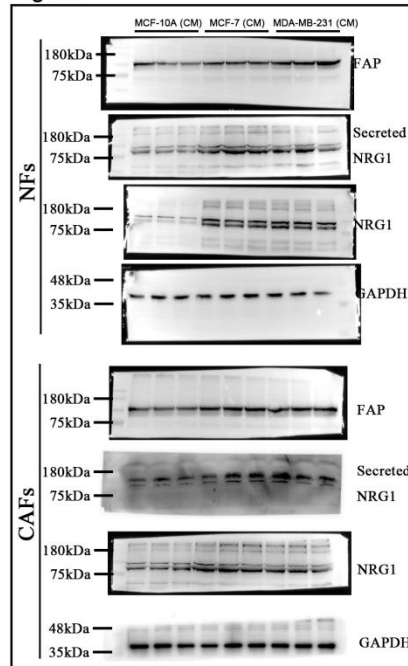

Fig 5B

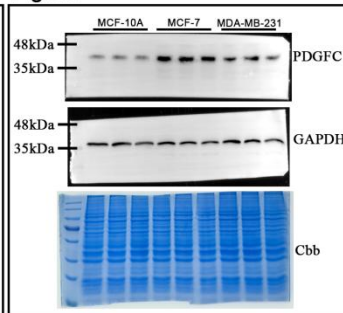

Fig 5F

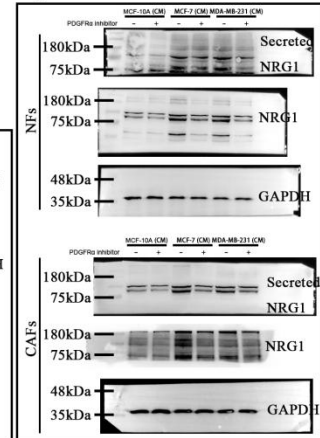

Fig 5E

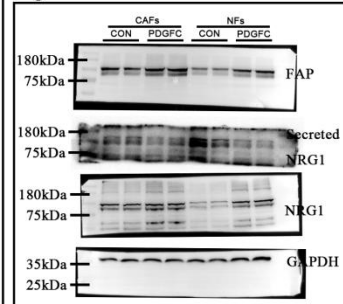

Fig 5G

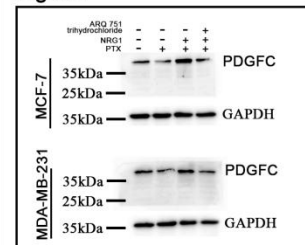

Fig S3A

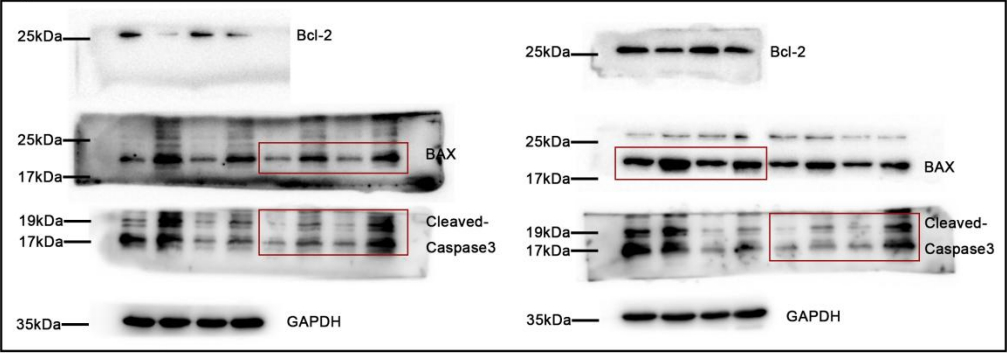

Fig S5A

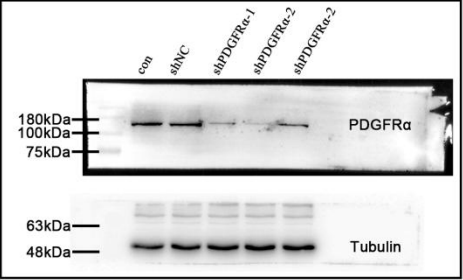

Fig S5C

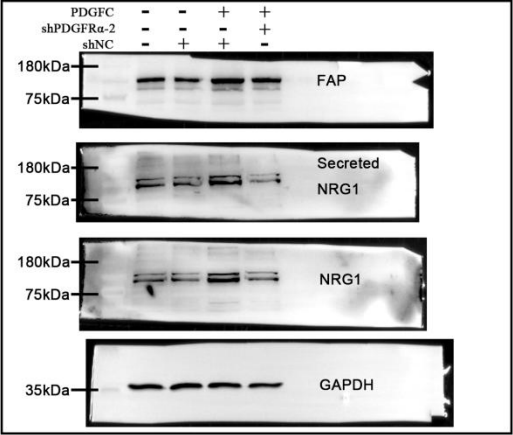

Supplement: Supplementary file 2 — Original data [file 41420_2025_2785_MOESM2_ESM.pdf]
